# Correction: Internalising and externalising behaviour in siblings of children born preterm Preterm birth: Internalising and externalising behaviour of siblings

**DOI:** 10.1371/journal.pmen.0000570

**Published:** 2026-02-26

**Authors:** Wnurinham Silva, Demetris Avraam, Luise Cederkvist, Johanna Lucia Nader, Maja Popovic, Hanan El Marroun, Jennifer R. Harris, Lorenzo Richiardi, Henning Tiemeier, Timothy James Cadman, Julia Jaekel, Anne-Marie Nybo Andersen, Eero Kajantie, Sylvain Sebert

The phrase ‘Preterm birth: Internalising and externalising behaviour of siblings’ should have not been included in the article title. The correct title is: Internalising and externalising behaviour in siblings of children born preterm. The correct citation is: Silva W, Avraam D, Cederkvist L, Nader JL, Popovic M, Marroun HE, et al. (2025) Internalising and externalising behaviour in siblings of children born preterm. PLOS Ment Health 2(6): e0000334. https://doi.org/10.1371/journal.pmen.0000334
