# Supplementary material for: Internalising and externalising behaviour in siblings of children born preterm Preterm birth: Internalising and externalising behaviour of siblings
Source: PLOS Ment Health. 2025 Jun 11;2(6):e0000334. doi: 10.1371/journal.pmen.0000334 (PMC12798436; doi:10.1371/journal.pmen.0000334)
Supplement: S1 Text — (DOCX) [file pmen.0000334.s001.docx]

# Cohorts’ information

## Participating cohort’s information

**The Norwegian Mother, Father and Child Cohort Study (MoBa)**

The Norwegian Mother, Father and Child Cohort Study (MoBa) MoBa is a Norwegian population-based longitudinal cohort study of around 114,500 children, 95,000 mothers and 75,000 fathers. Recruitment of pregnant women attending routine ultrasound examination took place between 1999 and 2009, and MoBa has since grown to become one of the largest population studies in the world. Baseline questionnaires were completed at around 15 weeks’ gestation, and two further prenatal questionnaires were administered at around 17-22 and 30 weeks’ gestation. In addition, fathers completed a questionnaire at around 15 weeks’ gestation. Follow-up questionnaires after birth were completed at 6 months, 18 months, 36 months, 5 years, 7 years, and 8 years of age, and data are currently being collected through questionnaires for children aged 13 and 14 years. The full cohort is being genotyped, with more than 33,000 participants (or 11,000 trios) genotyped to date. The purpose of MoBa is to improve our knowledge of diseases and health, by estimating exposure-outcome associations among children and their parents from preconception and pregnancy throughout life.

***Ethics approval***

The establishment and data collection in MoBa was previously based on a license from the Norwegian Data protection agency and approval from The Regional Committee for Medical Research Ethics, and it is now based on regulations related to the Norwegian Health Registry Act. MoBa is conducted according to the guidelines laid down in the declaration of Helsinki. The current study was approved by the Regional Committees for Medical and Health Research Ethics (REK Sør-Øst C: 2018/427).

***Consent to participate***

Written, informed consent for participating was obtained from the parent/guardian of each participant under 18 years of age prior to enrolment. A detailed protocol of the study including the consent can be found elsewhere (http://www.fhi.no/morogbarn).

**Danish National Birth Cohort (DNBC)**

The DNBC is a nation-wide conception-to-death study that includes the offspring of around 100,000 pregnancies. The pregnant women were enrolled in the cohort between 1996-2002 by signing an informed consent form handed out at the first antenatal care visit in general practice. Approximately 30% of the source population was included in the cohort. The women were invited for four telephone interviews: two pregnancy interviews (at around 16 and 30 weeks of gestation), and two post-partum interviews (at around child age of 6 and 18 months). The parent-child dyads were invited for follow-ups at child age of 7, 11, and at age 14 the children were asked to fill in a food frequency questionnaire. At age 18, the adolescents were invited for a follow-up.

***Ethics approval***

The DNBC complies with the Declaration of Helsinki and was approved by the Regional Scientific Ethical Committee for the Municipalities of Copenhagen and Frederiksberg - Danish National Committee on Biomedical Research Ethics- (Ref. no (KF) 01-471/94). Follow-ups have been approved according to Danish legislation.

***Consent to participate***

Written informed consent was obtained from participants upon enrolment.

**The Generation R Study (Gen R)**

The Generation R Study is a population-based prospective cohort study from fetal life until adulthood. The study is designed to identify early environmental and genetic causes and causal pathways leading to normal and abnormal growth, development and health from fetal life, childhood and young adulthood. This multidisciplinary study focuses on several health outcomes including behaviour and cognition, body composition, eye development, growth, hearing, heart and vascular development, infectious disease and immunity, oral health and facial growth, respiratory health, allergy and skin disorders of children and their parents. Main exposures of interest include environmental, endocrine, genomic (genetic, epigenetic, microbiome), lifestyle related, nutritional and socio-demographic determinants. In total, 9778 mothers with a delivery date from April 2002 until January 2006 were enrolled in the study. Response at baseline was 61%, and general follow-up rates until the age of 10 years were around 80%. Data collection in children and their parents includes questionnaires, interviews, detailed physical and ultrasound examinations, behavioural observations, lung function, Magnetic Resonance Imaging and biological sampling. Genome and epigenome wide association screens are available. Eventually, results from the Generation R Study contribute to the development of strategies for optimizing health and healthcare for pregnant women and children.

***Ethics approval***

The general design, all research aims and the specific measurements in the Generation R Study have been approved by the Medical Ethical Committee of the Erasmus Medical Center, Rotterdam. New measurements will only be embedded in the study after approval of the Medical Ethical Committee. The reference numbers of the ethical documents are as follows: phase 1 (fetal period) MEC 198.782/2001/31; phase 2 (0-4 years) MEC 217.595/2002/202; phase 3 (6 and 10 years) MEC-2007-413; MEC-2010-084; MEC-2012-165; phase 4 (13 and 17 years) MEC 2015-749).

***Consent to participate***

Participants are asked for their written informed consent for the four consecutive phases of the study (prenatally, birth to 4 years, 4–12 years, and from 12 years onwards). At the start of each phase, mothers and their partners receive written and oral information about the study. Even with consent of the parents, when the child is not willing to participate actively, no measurements are performed. From the age of 12 years, children are asked for written informed consent.

**Nascita ed INFanzia: gli Effetti dell'Ambiente (NINFEA)**

The NINFEA (Nascita ed INFanzia: gli Effetti dell'Ambiente) study is an Italian web-based birth cohort that recruited approximately 7500 pregnant women during the period 2005-2016. Members of the cohort are children of mothers who had access to the Internet, enough knowledge of Italian to complete online questionnaires, and volunteered to participate at any time during the pregnancy. At enrolment women completed the baseline questionnaire, and children are then followed up with seven questionnaires completed by mothers 6 and 18 months after delivery, and when children turn 4, 7, 10,13, and 16 years of age.

***Ethics approval***

The Ethical Committee of the San Giovanni Battista Hospital and CTO/CRF/Maria Adelaide Hospital of Turin approved the NINFEA study (approval N.0048362, and subsequent amendments).

***Consent to participate***

Written informed consent was obtained from all the participants at enrolment and at each follow-up.
