# Supplementary material for: Internalising and externalising behaviour in siblings of children born preterm Preterm birth: Internalising and externalising behaviour of siblings
Source: PLOS Ment Health. 2025 Jun 11;2(6):e0000334. doi: 10.1371/journal.pmen.0000334 (PMC12798436; doi:10.1371/journal.pmen.0000334)
Supplement: S2 Text — (DOCX) [file pmen.0000334.s002.docx]

Cohort-specific acknowledgments

**The Norwegian Mother, Father and Child Cohort Study (MoBa)**

The Norwegian Mother, Father and Child Cohort Study is supported by the Norwegian Ministry of Health and Care Services and the Ministry of Education and Research. We are grateful to all the participating families in Norway who take part in this on-going cohort study.

This work was partly supported by the Research Council of Norway through its Centres of Excellence funding scheme, project number **262700.**

**Danish National Birth Cohort (DNBC)**

The authors would like to thank the participants, the first Principal Investigator of DNBC Prof. Jørn Olsen, the scientific managerial team, and DNBC secretariat for being, establishing, developing and consolidating the Danish National Birth Cohort. The infrastructure and co-authors AMNA and LC were supported by EUCAN Connect.

**The Generation R Study (Gen R)**

The general design of the Generation R Study is made possible by financial support from Erasmus University Medical Center; Erasmus University Rotterdam; the Netherlands Organization for Health Research and Development; the Netherlands Organization for Scientific Research; the Ministry of Health, Welfare and Sport; and the Ministry of Youth and Families. The authors sincerely acknowledge the contributions of the participating children, parents, general practitioners, hospitals, midwives, and pharmacies in Rotterdam.

This work was supported by Stichting Volksbond Rotterdam (to Hanan El Marroun); the Netherlands Organization for Scientific Research Aspasia (Grant No. 015.016.056 [to Hanan El Marroun]), and the European Union’s Horizon 2020 Research and Innovation Program (HappyMums, Grant Agreement No. 101057390 [to Hanan El Marroun]) and the Netherlands Organisation for Health Research and Development (ZonMw Vici project No. 016.VICI.170.200 [to Henning Tiemeier]). The authors report no biomedical financial interests or potential conflicts of interest.

**Nascita ed INFanzia: gli Effetti dell'Ambiente (NINFEA)**

The authors thank all families participating in the NINFEA cohort.
