# Supplementary figures and images for: Internalising and externalising behaviour in siblings of children born preterm Preterm birth: Internalising and externalising behaviour of siblings
Source: PLOS Ment Health. 2025 Jun 11;2(6):e0000334. doi: 10.1371/journal.pmen.0000334 (PMC12798436; doi:10.1371/journal.pmen.0000334)

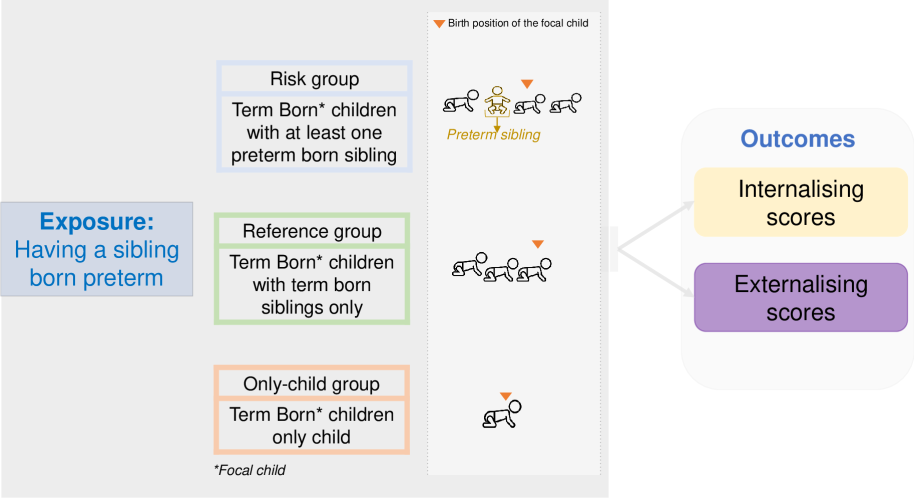

Supplement: S1 Fig — (TIF) [file pmen.0000334.s003.tif]
