# Supplementary material for: Internalising and externalising behaviour in siblings of children born preterm Preterm birth: Internalising and externalising behaviour of siblings
Source: PLOS Ment Health. 2025 Jun 11;2(6):e0000334. doi: 10.1371/journal.pmen.0000334 (PMC12798436; doi:10.1371/journal.pmen.0000334)
Supplement: S2 Fig — (DOCX) [file pmen.0000334.s004.docx]

# Across all ages analyses

**One and two-stage IPD meta-analyses across all ages**

**
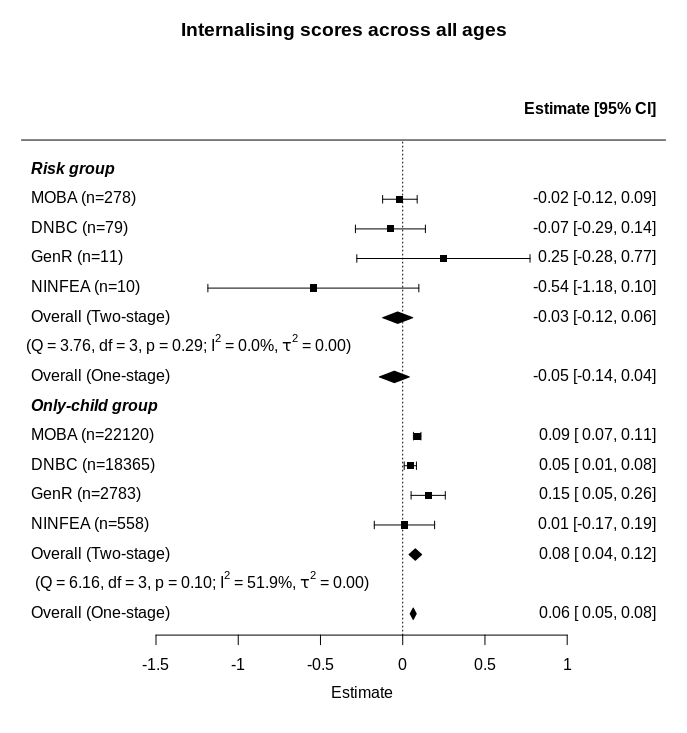
**

**Forest plot of association between having a sibling born preterm and internalising scores (z-score) across all ages.** We analysed average internalising scores from 0-14 years of age. Internalising scores (z-score) across all ages using two-stage and one-stage IPD meta-analyses adjusted models. Adjusted for age of the mother at birth, mother’s education level, pregnancy smoking and alcohol intake during pregnancy. Estimates indicate the difference in standardized internalising scores of children in risk and only-child groups compared to the scores of children in the reference group. The whiskers (95% CI), number of children included (next to cohort names), individual study estimates and overall estimates, *I*2 and *τ*2 statistics are presented for the two stage meta-analysis. For the one stage meta-analysis only the overall estimate and confidence interval are presented.


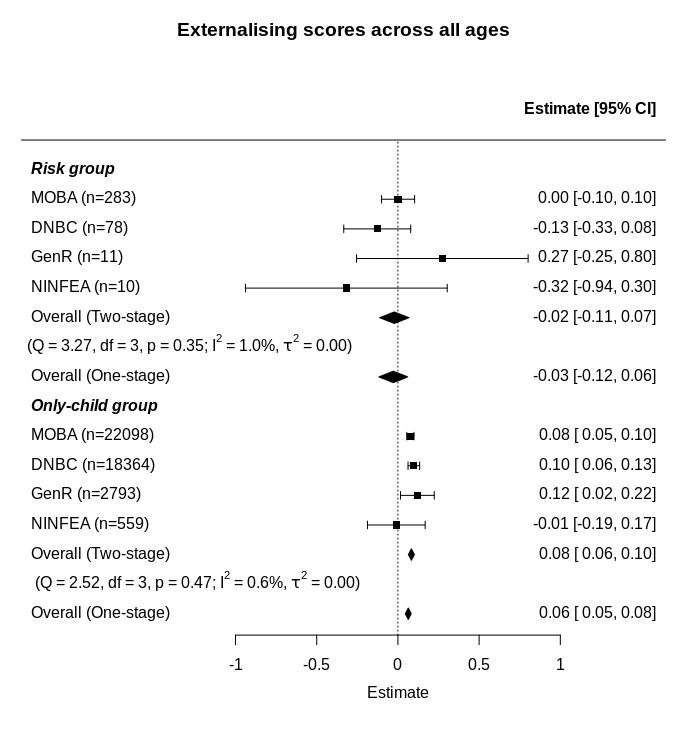


**Forest plot of association between having a sibling born preterm and externalising scores (z-score) across all ages.** We analysed average externalising scores from 0-14 years of age. Externalising scores (z-score) across all ages using two-stage and one-stage IPD meta-analyses adjusted models. Adjusted for age of the mother at birth, mother’s education level, pregnancy smoking and alcohol intake during pregnancy. Estimates indicate the difference in standardized externalising scores of children in risk and only-child groups compared to the scores of children in the reference group. The whiskers (95% CI), number of children included (next to cohort names), individual study estimates and overall estimates, *I*2 and *τ*2 statistics are presented for the two stage meta-analysis. For the one stage meta-analysis only the overall estimate and confidence interval are presented.

We found no evidence of association between having a sibling born preterm and increased risk of higher internalising and externalising behavioural in the risk group, from early childhood to early adolescence. However, in the only-child group, internalising and externalising scores were higher than the mean of the reference group in the two-stage and one-stage IPD meta-analyses.
