# Supplementary material for: Internalising and externalising behaviour in siblings of children born preterm Preterm birth: Internalising and externalising behaviour of siblings
Source: PLOS Ment Health. 2025 Jun 11;2(6):e0000334. doi: 10.1371/journal.pmen.0000334 (PMC12798436; doi:10.1371/journal.pmen.0000334)
Supplement: S1 Table — (DOCX) [file pmen.0000334.s005.docx]

Characteristics of the children in the risk group, reference group and only-child group with and without internalising and externalising scores

| **Cohort** | **MoBa (n=41,546)** | | | **DNBC (n=41,278)** | | | **GenR (n=4,752)** | | |
| --- | --- | --- | --- | --- | --- | --- | --- | --- | --- |
| **Variables** | Risk group  (n=344) | Reference group  (n=9,274) | Only-child group  (n=31,928) | Risk group  (n=149) | Reference group  (n=4,620) | Only-child group  (n=36,509) | Risk group  (n=13) | Reference group  (n=396) | Only-child group  (n=4,343) |
| Male | 172 (*50)* | 4,760 (*51)* | 16,358 (51) | 70 (*47)* | 2,370 (*51)* | 1,861 (51) | 5 (38) | 209 (53) | 2,150 (50) |
| Female | 172 (*50)* | 4,514 (*49)* | 15,570 (49) | 79 (*53)* | 2,250 (*49)* | 17,899 (49) | 8 (62) | 187 (47) | 2,193 (50) |
| NA | *-* | - | - | *-* | - | - | *-* | - | - |
|  |  |  |  |  |  |  |  |  |  |
| Focal GA, weeks: mean (SD) | 39.5 (1.3) | 40.3 (1.3) | 40.2 (1.3) | 39.6 (1.48) | 40.3 (1.30) | 40.2 (1.30) | 40.0 (1.6) | 40.7 (1.4) | 40.7 (1.4) |
|  |  |  |  |  |  |  |  |  |  |
| Sibling GA, weeks: mean (SD) | 34.6 (2.9) | 40.2 (1.2) | - | 34.9 (2.4) | 40.1 (1.2) | - | 34.1 (3.6) | 40.7 (1.1) | - |
|  |  |  |  |  |  |  |  |  |  |
| Maternal age, years: mean (SD) | 28.8 (4.3) | 28.2 (3.8) | 28.5 (4.6) | 28.7 (4.1) | 27.9 (3.5) | 28.2 (4.1) | 31.1 (3.8) | 30.4 (4.4) | 28.8 (5.4) |
|  |  |  |  |  |  |  |  |  |  |
| Maternal education: n (%) |  |  |  |  |  |  |  |  |  |
| High | 223 (65) | 6,309 (68) | 18,718 (59) | 61 (41) | 2,340 (51) | 17,194 (47) | 11(85) | 252 (64) | 1,694 (39) |
| Medium | 102 (30) | 2,298 (25) | 10,086 (32) | 30 (20) | 883 (19) | 6,979 (19) | Not shown* | 121 (31) | 1,935 (45) |
| Low | 7 (2) | 124 (1) | 976 (3) | 38 (26) | 989 (21) | 8,504 (23) | Not shown* | 17 (4) | 318 (7) |
| NA | 12 (3) | 543 (6) | 2,148 (7) | 20 (13) | 408 (9) | 3,832 (10) | - | 6 (2) | 396 (9) |
|  |  |  |  |  |  |  |  |  |  |
| Pregnancy smoking: n (%) |  |  |  |  |  |  |  |  |  |
| No | 249 (*72)* | 7,175 (*77)* | 23,167 (73) | 99 (*66)* | 3,482 (*75)* | 25,516 (70) | 7 (54) | 290 (73) | 2,717 (63) |
| Yes | 95 (*28)* | 2,099 (*23)* | 8,761 (27) | 44 (*30)* | 1,027 (*22)* | 10,194 (28) | 5 (38) | 67 (17) | 1,094 (25) |
| NA | - | - | - | 6 (*4)* | 111 (*2)* | 799 (2) | 1 (8) | 39 (10) | 532 (12) |
|  |  |  |  |  |  |  |  |  |  |
| Pregnancy alcohol intake: n (%) |  |  |  |  |  |  |  |  |  |
| No | 229 (*67)* | 5,826 (*62,8*) | 21,682 (68) | 54 (*36)* | 1,868 (*40)* | 16,225 (44) | Not shown* | 102 (26) | 1,757 (40) |
| Yes | 113 (*33)* | 3,428 (*37*) | 9,794 (31) | 89 (*60)* | 2,626 (*57)* | 19,361 (53) | 9 (69) | 249 (63) | 1,979 (46) |
| NA | 2 (*1)* | 20 (*0.2)* | 452 (1) | 6 (*4)* | 126 (*3)* | 923 (3) | 2 (15) | 45 (11) | 607 (14) |

S3. Continuation

| **Cohort** | **NINFEA (n=4,239)** | | | **All cohorts (n=91,815)** | | |
| --- | --- | --- | --- | --- | --- | --- |
| **Variables** | Risk group  (n=23) | Reference group  (n=509) | Only-child group  (n=3,707) | Risk group  (n=529) | Reference group  (n=14,799) | Only-child group  (n=76,487) |
| Male | 9 (*39)* | 272 (*53)* | 1,860 (50) | 256 (*48)* | 7,611 (*51)* | 38,978 (51) |
| Female | 14 (*61)* | 236 (*46)* | 1,794 (48) | 273 (*52)* | 7,187 (*49)* | 37,456 (49) |
| NA | *-* | 1 (*0,4)* | 53 (1) | *-* | 1 (*0,01)* | 53 (0.1) |
|  |  |  |  |  |  |  |
| Focal GA, weeks: mean (SD) | 39.2 (1.1) | 40.0 (1.2) | 40.0 (1.3) | 39.54 (1.4) | 40.3 (1.3) | 40.2 (1.3) |
|  |  |  |  |  |  |  |
| Sibling GA, weeks: mean (SD) | 35.7 (1.5) | 39.9 (1.2) | - | 34.7 (2.8) | 40.2 (1.2) | - |
|  |  |  |  |  |  |  |
| Maternal age, years: mean (SD) | 31.7 (3.1) | 31.2 (3.5) | 32.6 (4.3) | 28.9 (4.2) | 28.3 (3.7) | 28.6 (4.4) |
|  |  |  |  |  |  |  |
| Maternal education: n (%) |  |  |  |  |  |  |
| High | 15 (*65)* | 350 (69) | 2,309 (62) | 299 (57) | 9,251 (63) | 39,915 (52) |
| Medium | 7 (*30)* | 142 (28) | 1,211 (33) | 139 (26) | 3,444 (23) | 20,211 (26) |
| Low | 1 (*4)* | 16 (3) | 160 (4) | 46 (9) | 1,146 (8) | 9,958 (13) |
| NA | - | 1 (0,2) | 27 (1) | 32 (6) | 958 (6) | 6,403 (8) |
|  |  |  |  |  |  |  |
| Pregnancy smoking: n (%) |  |  |  |  |  |  |
| No | 23 (*100)* | 472 (*93)* | 3,317 (89) | 378 (*71)* | 11,419 (*77)* | 54,717 (72) |
| Yes | - | 34 (*7)* | 344 (9) | 144 (*27)* | 3,227 (*22)* | 20,393 (27) |
| NA | - | 3 (*1)* | 46 (1) | 7 (*1)* | 153 (*1)* | 1,377 (2) |
|  |  |  |  |  |  |  |
| Pregnancy alcohol intake: n (%) |  |  |  |  |  |  |
| No | 20 (*87)* | 327 (*64)* | 2,391 (64) | 305 (*58)* | 8,123 (*55)* | 42,055 (55) |
| Yes | 3 (*13)* | 167 (*33)* | 1,001 (27) | 214 (*40)* | 6,470 (*44)* | 32,135 (42) |
| NA | - | 15 (*3)* | 315 (8) | 10 (*2)* | 206 (*1)* | 2,297 (3) |

Focal children: term-born children (≥ 37 weeks GA) for whom who were interested analysing behaviour problems. Table includes total number of children by group after all exclusion criteria, but including those with and without internalising and externalising behaviour measurements;

Groups: risk group (term-born children with preterm-born sibling(s)); reference group (term-born children with term-born sibling(s)); only-child group (term-born children without siblings);

* Fewer than 3 in the summary statistic so value has not been presented owing to risk of disclosure;

Abbreviations: GA: gestational age; MoBa: Norwegian Mother, Father and Child Cohort Study in Norway; DNBC: Danish National Birth Cohort in Denmark; GenR: the Generation R Study; NINFEA: Nascita e INFanzia: gli Effetti dell’Ambiente.
