# Supplementary material for: Internalising and externalising behaviour in siblings of children born preterm Preterm birth: Internalising and externalising behaviour of siblings
Source: PLOS Ment Health. 2025 Jun 11;2(6):e0000334. doi: 10.1371/journal.pmen.0000334 (PMC12798436; doi:10.1371/journal.pmen.0000334)
Supplement: S2 Table — (DOCX) [file pmen.0000334.s006.docx]

# Cohort specific estimates

## Linear regressions by cohort and by age group ( ≤4 ; 5-10 ; 11-14)

For each cohort, separate tables are provided for internalising and externalising behaviours estimates and for each of the behaviours crude and adjusted models are provided in separate tables:

Internalising behaviour crude (A.1)

Internalising behaviour adjusted (B.1)

Externalising behaviour crude (C.1)

Externalising behaviour adjusted (D.1)

All adjusted models have been adjusted for age of the mother at birth, pregnancy smoking, pregnancy alcohol and Mother’s education level. Tables show the number of focal children per group, regression coefficients, 95%CI and p-values.

**MoBa (A.1)**

| Age group | Group | Nr of Focal children/group | Regression coefficient | 95%CI | P-value |
| --- | --- | --- | --- | --- | --- |
| Age ≤4 | Reference | 8198 |  |  |  |
|  | Risk group | 291 | -0.060 | -0.181 , 0.060 | 0.326 |
|  | Only-child group | 23202 | 0.068 | 0.042, 0.094 | <0.001 |
| Age 5-10 | Reference | 1196 |  |  |  |
|  | Risk group | 42 | 0.411 | 0.093 , 0.729 | 0.011 |
|  | Only-child group | 10288 | 0.115 | 0.053 , 0.177 | <0.001 |
| Age 11-14 | Reference |  |  |  |  |
|  | Risk group |  |  |  |  |
|  | Only-child group |  |  |  |  |

**MoBa (B.1)**

| Age group | Group | Nr of Focal children/group | Regression coefficient | 95%CI | P-value |
| --- | --- | --- | --- | --- | --- |
| Age ≤4 | Reference | 7710 |  |  |  |
|  | Risk group | 277 | -0.081 | -0.204 , 0.041 | 0.196 |
|  | Only-child group | 21659 | 0.058 | 0.031 , 0.085 | <0.001 |
| Age 5-10 | Reference | 1133 |  |  |  |
|  | Risk group | 39 | 0.424 | 0.096 , 0.752 | 0.011 |
|  | Only-child group | 9642 | 0.101 | 0.038 , 0.164 | 0.0016 |
| Age 11-14 | Reference |  |  |  |  |
|  | Risk group |  |  |  |  |
|  | Only-child group |  |  |  |  |

**MoBa (C.1)**

| Age group | Group | Nr of Focal children/group | Regression coefficient | 95%CI | P-value |
| --- | --- | --- | --- | --- | --- |
| Age ≤4 | Reference | 8155 |  |  |  |
|  | Risk group | 295 | 0.053 | -0.062 , 0.168 | 0.368 |
|  | Only-child group | 22978 | 0.069 | 0.044 , 0.094 | <0.001 |
| Age 5-10 | Reference | 3671 |  |  |  |
|  | Risk group | 133 | -0.101 | -0.275 , 0.071 | 0.250 |
|  | Only-child group | 13457 | 0.128 | 0.092 , 0.165 | <0.001 |
| Age 11-14 | Reference |  |  |  |  |
|  | Risk group |  |  |  |  |
|  | Only-child group |  |  |  |  |

**MoBa (D.1)**

| Age group | Group | Nr of Focal children/group | Regression coefficient | 95%CI | P-value |
| --- | --- | --- | --- | --- | --- |
| Age ≤4 | Reference | 7670 |  |  |  |
|  | Risk group | 281 | 0.021 | -0.095 , 0.138 | 0.721 |
|  | Only-child group | 21451 | 0.056 | 0.030 , 0.082 | <0.001 |
| Age 5-10 | Reference | 3474 |  |  |  |
|  | Risk group | 128 | -0.082 | -0.257 , 0.092 | 0.356 |
|  | Only-child group | 12592 | 0.111 | 0.074 , 0.149 | <0.001 |
| Age 11-14 | Reference |  |  |  |  |
|  | Risk group |  |  |  |  |
|  | Only-child group |  |  |  |  |

**DNBC (A.1)**

| Age group | Group | Nr of Focal children/group | Regression coefficient | 95%CI | P-value |
| --- | --- | --- | --- | --- | --- |
| Age ≤4 | Reference |  |  |  |  |
|  | Risk group |  |  |  |  |
|  | Only-child group |  |  |  |  |
| Age 5-10 | Reference | 3052 |  |  |  |
|  | Risk group | 91 | -0.115 | -0.330 , 0.100 | 0.294 |
|  | Only-child group | 21430 | 0.030 | -0.008 , 0.069 | 0.127 |
| Age 11-14 | Reference | 2627 |  |  |  |
|  | Risk group | 73 | -0.163 | -0.399 , 0.071 | 0.172 |
|  | Only-child group | 17944 | 0.084 | 0.043 , 0.126 | <0.001 |

**DNBC (B.1)**

| Age group | Group | Nr of Focal children/group | Regression coefficient | 95%CI | P-value |
| --- | --- | --- | --- | --- | --- |
| Age ≤4 | Reference |  |  |  |  |
|  | Risk group |  |  |  |  |
|  | Only-child group |  |  |  |  |
| Age 5-10 | Reference | 2539 |  |  |  |
|  | Risk group | 68 | -0.063 | -0.309 , 0.182 | 0.613 |
|  | Only-child group | 15905 | 0.028 | -0.013 , 0.071 | 0.185 |
| Age 11-14 | Reference | 2165 |  |  |  |
|  | Risk group | 54 | -0.172 | -0.443 , 0.099 | 0.213 |
|  | Only-child group | 13446 | 0.077 | 0.031 , 0.122 | <0.001 |

**DNBC (C.1)**

| Age group | Group | Nr of Focal children/group | Regression coefficient | 95%CI | P-value |
| --- | --- | --- | --- | --- | --- |
| Age ≤4 | Reference |  |  |  |  |
|  | Risk group |  |  |  |  |
|  | Only-child group |  |  |  |  |
| Age 5-10 | Reference | 3052 |  |  |  |
|  | Risk group | 90 | -0.076 | -0.284 , 0.131 | 0.470 |
|  | Only-child group | 21419 | 0.095 | 0.057 , 0.132 | <0.001 |
| Age 11-14 | Reference | 2627 |  |  |  |
|  | Risk group | 73 | -0.192 | -0.420 , 0.035 | 0.098 |
|  | Only-child group | 17944 | 0.160 | 0.120 , 0.200 | <0.001 |

**DNBC (D.1)**

| Age group | Group | Nr of Focal children/group | Regression coefficient | 95%CI | P-value |
| --- | --- | --- | --- | --- | --- |
| Age ≤4 | Reference |  |  |  |  |
|  | Risk group |  |  |  |  |
|  | Only-child group |  |  |  |  |
| Age 5-10 | Reference | 2539 |  |  |  |
|  | Risk group | 67 | -0.100 | -0.335 , 0.134 | 0.400 |
|  | Only-child group | 15901 | 0.071 | 0.030 , 0.111 | <0.001 |
| Age 11-14 | Reference | 2165 |  |  |  |
|  | Risk group | 54 | -0.145 | -0.402 , 0.111 | 0.268 |
|  | Only-child group | 13446 | 0.126 | 0.082 , 0.169 | <0.001 |

**GenR (A.1)**

| Age group | Group | Nr of Focal children/group | Regression coefficient | 95%CI | P-value |
| --- | --- | --- | --- | --- | --- |
| Age ≤4 | Reference | 327 |  |  |  |
|  | Risk group | 12 | -0.249 | -0.825 , 0.327 | 0.396 |
|  | Only-child group | 2543 | 0.061 | -0.054 , 0.176 | 0.299 |
| Age 5-10 | Reference | 327 |  |  |  |
|  | Risk group | 8 | 0.360 | -0.383 , 1.103 | 0.342 |
|  | Only-child group | 2926 | 0.254 | 0.133 , 0.375 | <0.001 |
| Age 11-14 | Reference |  |  |  |  |
|  | Risk group |  |  |  |  |
|  | Only-child group |  |  |  |  |

**GenR (B.1)**

| Age group | Group | Nr of Focal children/group | Regression coefficient | 95%CI | P-value |
| --- | --- | --- | --- | --- | --- |
| Age ≤4 | Reference | 288 |  |  |  |
|  | Risk group | 11 | -0.127 | -0.717 , 0.463 | 0.673 |
|  | Only-child group | 2141 | 0.062 | -0.059 , 0.183 | 0.316 |
| Age 5-10 | Reference | 282 |  |  |  |
|  | Risk group | 7 | 0.298 | -0.490 , 1.087 | 0.458 |
|  | Only-child group | 2489 | 0.265 | 0.134 , 0.396 | <0.001 |
| Age 11-14 | Reference |  |  |  |  |
|  | Risk group |  |  |  |  |
|  | Only-child group |  |  |  |  |

**GenR (C.1)**

| Age group | Group | Nr of Focal children/group | Regression coefficient | 95%CI | P-value |
| --- | --- | --- | --- | --- | --- |
| Age ≤4 | Reference | 326 |  |  |  |
|  | Risk group | 12 | -0.299 | -0.883 , 0.284 | 0.314 |
|  | Only-child group | 2555 | 0.110 | -0.006 , 0.227 | 0.063 |
| Age 5-10 | Reference | 326 |  |  |  |
|  | Risk group | 8 | 0.628 | -0.091 , 1.348 | 0.086 |
|  | Only-child group | 2952 | 0.192 | 0.075 , 0.310 | 0.0012 |
| Age 11-14 | Reference |  |  |  |  |
|  | Risk group |  |  |  |  |
|  | Only-child group |  |  |  |  |

**GenR (D.1)**

| Age group | Group | Nr of Focal children/group | Regression coefficient | 95%CI | P-value |
| --- | --- | --- | --- | --- | --- |
| Age ≤4 | Reference | 286 |  |  |  |
|  | Risk group | 11 | -0.200 | -0.808 , 0.407 | 0.518 |
|  | Only-child group | 2153 | 0.102 | -0.022 , 0.227 | 0.108 |
| Age 5-10 | Reference | 282 |  |  |  |
|  | Risk group | 7 | 0.825 | 0.055 , 1.596 | 0.035 |
|  | Only-child group | 2509 | 0.196 | 0.068 , 0.324 | 0.002 |
| Age 11-14 | Reference |  |  |  |  |
|  | Risk group |  |  |  |  |
|  | Only-child group |  |  |  |  |

**NINFEA (A.1)**

| Age group | Group | Nr of Focal children/group | Regression coefficient | 95%CI | P-value |
| --- | --- | --- | --- | --- | --- |
| Age ≤4 | Reference |  |  |  |  |
|  | Risk group |  |  |  |  |
|  | Only-child group |  |  |  |  |
|  | Reference |  |  |  |  |
|  | Risk group |  |  |  |  |
|  | Only-child group |  |  |  |  |
| Age 11-14 | Reference | 151 |  |  |  |
|  | Risk group | 10 | -0.558 | -1.203 , 0.085 | 0.089 |
|  | Only-child group | 570 | -0.019 | -0.200 , 0.161 | 0.831 |

**NINFEA (B.1)**

| Age group | Group | Nr of Focal children/group | Regression coefficient | 95%CI | P-value |
| --- | --- | --- | --- | --- | --- |
| Age 5-10 | Reference |  |  |  |  |
|  | Risk group |  |  |  |  |
|  | Only-child group |  |  |  |  |
|  | Reference |  |  |  |  |
| Age 5-10 | Reference |  |  |  |  |
|  | Risk group |  |  |  |  |
|  | Only-child group |  |  |  |  |
| Age 11-14 | Reference | 145 |  |  |  |
|  | Risk group | 10 | -0.543 | -1.184 , 0.098 | 0.096 |
|  | Only-child group | 558 | 0.010 | -0.172 , 0.193 | 0.910 |

**NINFEA (C.1)**

| Age group | Group | Nr of Focal children/group | Regression coefficient | 95%CI | P-value |
| --- | --- | --- | --- | --- | --- |
| Age ≤4 | Reference |  |  |  |  |
|  | Risk group |  |  |  |  |
|  | Only-child group |  |  |  |  |
| Age 5-10 | Reference |  |  |  |  |
|  | Risk group |  |  |  |  |
|  | Only-child group |  |  |  |  |
| Age 11-14 | Reference | 151 |  |  |  |
|  | Risk group | 10 | -0.352 | -0.967 , 0.263 | 0.261 |
|  | Only-child group | 571 | -0.036 | -0.208 , 0.136 | 0.681 |

**NINFEA (D.1)**

| Age group | Group | Nr of Focal children/group | Regression coefficient | 95%CI | P-value |
| --- | --- | --- | --- | --- | --- |
| Age 5-10 | Reference |  |  |  |  |
|  | Risk group |  |  |  |  |
|  | Only-child group |  |  |  |  |
| Age 5-10 | Reference |  |  |  |  |
|  | Risk group |  |  |  |  |
|  | Only-child group |  |  |  |  |
| Age 11-14 | Reference | 145 |  |  |  |
|  | Risk group | 10 | -0.317 | -0.938 , 0.303 | 0.316 |
|  | Only-child group | 559 | -0.009 | -0.187 , 0.167 | 0.913 |
