# Supplementary material for: Internalising and externalising behaviour in siblings of children born preterm Preterm birth: Internalising and externalising behaviour of siblings
Source: PLOS Ment Health. 2025 Jun 11;2(6):e0000334. doi: 10.1371/journal.pmen.0000334 (PMC12798436; doi:10.1371/journal.pmen.0000334)
Supplement: S3 Table — (DOCX) [file pmen.0000334.s007.docx]

# One-stage IPD meta-analysis estimates

## One-stage by age group (≤4; 5-10 ; 11-14)

In one stage IPD meta-analysis the results were generated as if the data from all cohorts were transferred to a central environment and analysed jointly. Analyses have been adjusted by cohort effect.

Tables are provided separately for internalising and externalising behaviours and for each of the behaviours crude and adjusted models are provided in separate tables:

Internalising behaviour crude (A.3)

Internalising behaviour adjusted (B.3)

Externalising behaviour crude (C.3)

Externalising behaviour adjusted (D.3)

All adjusted models have been adjusted for age of the mother at birth, pregnancy smoking, pregnancy alcohol and Mother’s education level. Tables show the number of index children per group, regression coefficients, 95%CI and p-values.

**One-stage meta-analysis (A.3)**

| Age group | Group | Nr of Focal children/group | Regression coefficient | 95%CI | P-value |
| --- | --- | --- | --- | --- | --- |
| Age ≤4 | Reference | 11071 |  |  |  |
|  | Risk group | 377 | -0.068 | -0.186 , 0.050 | 0.259 |
|  | Only-child group | 42726 | 0.068 | 0.042 , 0.093 | <0.001 |
| Age 5-10 | Reference | 4720 |  |  |  |
|  | Risk group | 151 | 0.068 | -0.104 , 0.242 | 0.437 |
|  | Only-child group | 35202 | 0.069 | 0.037 , 0.101 | <0.001 |
| Age 11-14 | Reference | 4301 |  |  |  |
|  | Risk group | 133 | -0.205 | -0.426 , 0.015 | 0.067 |
|  | Only-child group | 31728 | 0.079 | 0.039 , 0.119 | <0.001 |

**One-stage meta-analysis (B.3)**

| Age group | Group | Nr of Focal children/group | Regression coefficient | 95%CI | P-value |
| --- | --- | --- | --- | --- | --- |
| Age ≤4 | Reference | 10455 |  |  |  |
|  | Risk group | 361 | -0.082 | -0.203 , 0.037 | 0.176 |
|  | Only-child group | 41449 | 0.059 | 0.033 , 0.085 | <0.001 |
| Age 5-10 | Reference | 4423 |  |  |  |
|  | Risk group | 138 | 0.120 | -0.070 , 0.311 | 0.215 |
|  | Only-child group | 32333 | 0.064 | 0.029 , 0.098 | <0.001 |
| Age 11-14 | Reference | 3961 |  |  |  |
|  | Risk group | 120 | -0.224 | -0.474 , -0.024 | 0.077 |
|  | Only-child group | 29112 | 0.073 | 0.028 , 0.117 | <0.001 |

**One-stage meta-analysis (C.3)**

| Age group | Group | Nr of Focal children/group | Regression coefficient | 95%CI | P-value |
| --- | --- | --- | --- | --- | --- |
| Age ≤4 | Reference | 10789 |  |  |  |
|  | Risk group | 373 | 0.039 | -0.074 , 0.152 | 0.498 |
|  | Only-child group | 41801 | 0.071 | 0.046 , 0.095 | <0.001 |
| Age 5-10 | Reference | 7194 |  |  |  |
|  | Risk group | 241 | -0.067 | -0.197 , 0.063 | 0.315 |
|  | Only-child group | 38387 | 0.116 | 0.090 , 0.142 | <0.001 |
| Age 11-14 | Reference | 6454 |  |  |  |
|  | Risk group | 214 | -0.201 | -0.415 , 0.012 | 0.065 |
|  | Only-child group | 34058 | 0.150 | 0.111, 0.189 | <0.001 |

**One-stage meta-analysis (D.3)**

| Age group | Group | Nr of Focal children/group | Regression coefficient | 95%CI | P-value |
| --- | --- | --- | --- | --- | --- |
| Age ≤4 | Reference | 11399 |  |  |  |
|  | Risk group | 398 | 0.013 | -0.101 , 0.128 | 0.820 |
|  | Only-child group | 46987 | 0.059 | 0.034 , 0.085 | <0.001 |
| Age 5-10 | Reference | 6758 |  |  |  |
|  | Risk group | 226 | -0.064 | -0.202 , 0.072 | 0.356 |
|  | Only-child group | 35280 | 0.098 | 0.071 , 0.125 | <0.001 |
| Age 11-14 | Reference | 6064 |  |  |  |
|  | Risk group | 201 | -0.163 | -0.399 , 0.073 | 0.176 |
|  | Only-child group | 31369 | 0.118 | 0.076 , 0.160 | <0.001 |
