# Supplementary material for: Internalising and externalising behaviour in siblings of children born preterm Preterm birth: Internalising and externalising behaviour of siblings
Source: PLOS Ment Health. 2025 Jun 11;2(6):e0000334. doi: 10.1371/journal.pmen.0000334 (PMC12798436; doi:10.1371/journal.pmen.0000334)
Supplement: Attachment — Submitted filename: Response_Letter 231024.docx [file pmen.0000334.s009.docx]

Dear Editor,

We would like to thank the three reviewers for their thoughtful comments and their suggestions that have helped us to improve our paper. We have revised the manuscript carefully in response to their comments; in what follows, we respond to each of the reviewers’ comments and describe the changes introduced in the revised manuscript.

We hope that the responses provided in this document and in the manuscript positively address the reviewers’ comments and suggestions. However, please do not hesitate to contact us should you have any questions or further suggestions.

Kind regards,

Wnurinham Silva (on behalf of the authors team)

Note: Line numbers refer to the clean version of the manuscript.

Reviewer #1:

QR1-1: General: The authors mix present and past tenses throughout the manuscript (e.g., lines 167 – 172). Please edit and be consistent.

**Response:** Thanks for the comment. The whole manuscript has been reviewed and edited where necessary.

QR1-2: Lines 93-95: What are the short- and long-term impacts of preterm birth on parental stress and depression/mental health? More details are needed.

**Response:** We agree and have included the following text in the revised manuscript (lines 95-97):

Families with children born preterm (before 37 weeks of gestation) may experience changes in their home environment stemming from the health care needs of the preterm child. Short- and long-term impacts of preterm birth on parents, such as stress and depression, have been extensively studied (10–12). For instance, it has been found that family stress levels remain elevated seven years after the birth of a preterm child (11). *Such changes in mental health and psychosocial stress may impact parenting, which, in turn, may affect the children’s behaviour.* Thus, it can be hypothesised that siblings may indirectly be affected by having a preterm born sibling.

QR1-3: Lines 189-191: The term “null parity” needs to be explained.

**Response:** Thanks. We have added an explanation of the term in the revised manuscript (lines 191-194):

*Hence, we included only firstborn children whose mothers had null parity, defined as no previous children at the time of the first birth in our dataset, to ensure that there are no previous exposures to circumstances related to siblings.*

QR1-4: Figure 2: Unnecessary as the details are explained in the manuscript. I suggest putting it into the supplement.

**Response:** Thanks for the suggestion. This has been moved to supplementary materials.

QR1-5: Lines 206-211: CBCL and MFQ should be explained in more detail: what do they measure, which (sub)scales were used, etc. Also, details for the calculation of the z scores need to be provided.

**Response:** We have provided more details about the two instruments (CBCL and SDQ) and about the calculation of the z-scores. In the revised manuscript (lines 204-225) we now have the following text:

*Internalising and externalising behaviours were measured using one of two well-established parent-report instruments: the Strengths and Difficulties Questionnaire (SDQ; DNBC and NINFEA) (29), or the Child Behaviour Checklist (CBCL; GenR and MoBa) (30). The SDQ contains 25‐items measured on a 3‐point Likert scale (“not true”, “somewhat true”, “certainly true”) containing five subscales: Emotional Problems, Conduct Problems, Hyperactivity, Peer Problems, and Pro‐social behaviour. Internalising scores are calculated by combining the Emotional Problems and Peer Problems subscales, while externalising scores are calculated by combining the Conduct Problems and Hyperactivity subscales. The CBCL contains 99‐items rated on a 3‐point Likert scale (“not true”, “sometimes true”, “often true”), containing eight subscales: Delinquent Behavior, Aggressive Behavior, Withdrawn, Somatic Complaints, Anxious/Depressed, Social Problems, Thought Problems, and Attention Problems. Internalising scores are calculated by combining the Withdrawn, Somatic Complaints, and Anxious/Depressed subscales, while externalising scores are calculated by combining the Delinquent and Aggressive Behaviours.*

*To allow statistical comparability across instruments we z-standardized the internalising and externalising scores by age according to the term born children’s scores within each cohort before the main analysis. Z-scores have been calculated by subtracting from each individual raw score (internalising or externalising) the mean value and dividing by the standard deviation of the raw scores of all term born children in the cohort. This transformation standardises the scores to a distribution with mean equal to 0 and standard deviation equal to 1. This helps overcome scaling differences across the harmonised dimensional scores within the respective age-bands of the CBCL and SDQ.*

QR1-6: Lines 222-226: Details are needed how maternal education was divided into high, medium, and low (e.g., based on which school degree, school years, or similar). Also, why was maternal mental health not considered as a confounder? Research has shown that maternal mental health has significant impact on children's mental health and behaviour.

**Response:** We provided more details about maternal education. We also agree that maternal mental health may have an effect on children's mental health and behaviour via genetic and environmental pathways. However, we were not able to include this variable as a confounder due to the high proportion of missing values in this variable across the participating cohorts. In the revised manuscript (lines 240-249) we include the following text:

*Maternal educational level was harmonized across cohorts based on the International Standard Classification of Education 97 (ISCED‐97) and consisted of three categories: Low (No education to lower secondary; ISCED‐97 categories 0‐2), Medium (Upper and post‐secondary; ISCED‐97 categories 3‐4), High (Degree and above; ISCED‐97 categories 5‐6). Despite evidence of the impact of maternal mental health on internalising and externalising behaviour of children we have not been able to include such variable as a confounder due to a high proportion of missing values. The harmonised data indeed include an indicator for any exposure to relatives such as parents or other individuals close to the child with mental disorders, however the proportion of missing values in this yearly repeated variable ranged from 67.3% in DNBC to 100% in MoBa.*

QR1-7: Lines 246-249: This approach is unclear: why was it done? What exactly was done? More details are needed.

**Response:** We have provided more details about the statistical approaches used. We have included the following text in the revised manuscript (lines 258- 280):

*We conducted a two-stage individual participant data (IPD) meta-analysis. With this approach, a regression model is fitted in each cohort separately and then the cohort-specific estimates and standard errors are combined with random effects meta-analysis. We used the rma function from the metafor R package (version 4.6-0) with the Restricted Estimate Maximum Likelihood method for the random-effects meta-analysis. With this method, the combined estimate is given as the weighted average of the cohort-specific estimates where the weights are defined as the inverse of the variance of the estimates. Between-cohort heterogeneity was evaluated with two-stage IPD meta-analysis using I-squared (I^2) and chi-squared Q-statistics. The analysis was conducted by age groups (≤ 4 years, 5–10 years, 11–14 years).*

*We performed complete case analysis, including participants with at least one internalising and externalising score available. Unadjusted models and models adjusted for confounders were used. The regression estimates can be interpreted as the mean difference in internalising and externalising z-scores for a group taking the Reference group as a reference. Cohort specific estimates in separate age groups are provided as supporting information.*

*Finally, we conducted sensitivity analyses to explore whether associations remained, by applying (i) one-stage IPD analysis adjusting the models by cohort indicators, and (ii) “across all ages” analyses using individual average internalising and externalising scores from age 0 to 14 years.*

*The one-stage IPD analysis is designed to give similar results as a pooled analysis where all the data could be combined and analysed jointly. DataSHIELD allows this method in an iteration process where no individual-level information is transferred or shared between the participating cohorts, but only non-disclosive summary statistics (e.g. score vectors and information matrices) (The procedure is described in 35).*

QR1-8: Table 2: The Sex NA row is not necessary as it has no entries. Focal birth weight is listed but not explained why it is relevant and what the norms for birth weight are.

**Response:** The sex NA row has been removed from the table. We have also removed the row with focal birth weight since this variable is not used in our analysis.

QR1-9: Lines 322-356: Meta-analysis results are available in the figure but missing in the text! Please include the results (MD, 95% CI, p) in the text to avoid confusion. High heterogeneity was found for some results, but no analysis was provided to further examine it.

**Response:** Thank you. The results of the two-stage meta-analysis have been added to the text as recommended (lines 358, 381).

Regarding the heterogeneity, indeed it could have been explored through meta-regression. However, there are some barriers that blocked us from doing meta-regression. As also described in our response to comment 18, the first barrier is the small number of studies. We only have 4 studies and because we divided our analysis into three age-groups we have data from only two studies in two of the age-groups and data from three studies in the third age-group. There is a rule of thumb that meta-regression should generally not be considered when there are fewer than ten studies in a meta-analysis (e.g. <https://handbook-5-1.cochrane.org/chapter_9/9_6_4_meta_regression.htm>). The small number of studies also blocked us from doing a “leave-one-out” sensitivity analysis. In addition to the small number of studies, we also have a small number of data in the only-child group for which we get high heterogeneity for the model with internalising behaviour at the age-group 5-10 years. The small number of data might produce biased results in the regressions, as we also discussed in the paper, and this might also lead to biased results in meta-analysis and further in meta-regression. The third barrier is the unavailability of specific study-level variables that could be used as potential effect modifiers in a meta-regression. The unavailability is related to multiple reasons including that cohorts might not collected data on such variables or might not harmonised such data for federated analyses. Also, for this project we got a remote access (through DataSHIELD) to a specific set of variables that we included in a project proposal, approved initially by the EU Child Cohort Network and then by the participating cohorts. Access to additional variables is therefore a long-time process.

For all these limitations we therefore decided to report the indicators of heterogeneity (e.g. I squared values) but to not explore its possible factors. We hope that the reviewer understands these barriers. We further expanded the text for the limitations of our study in the discussion section (lines 498-502):

*The heterogeneity observed in the population is potentially created by the age differences within and across the cohorts, in addition to other characteristics. However, we have not been able to further examine the heterogeneity, for example using meta-regression, due to the small number of cohorts and to the unavailability of cohort-level variables.*

QR1-10: Figures 2-4: Captions are too long, consider shortening them. Further, it needs to be explained what the positive and negative estimates in the forest plots represent. Does a positive estimate mean “no difference between the groups”? Renaming the x axis title “adjusted estimate” to something that better explains the outcome could help avoiding this problem.

**Response:** Many thanks for the comment. We have shortened the descriptions as much as possible and explained the meaning of the positive or negative estimates in the forest plots. Additionally, we have changed the title of the x axis from “adjusted estimate” to “Mean difference”.

QR1-11: Lines 376-377: Increased or decreased scores? The children had scores but how did they differ (or not differ) in their scores compared to the reference group?

**Response:** Thanks for the comment. We have rephrased this in the revised manuscript (lines 407-409):

*We found no evidence that children with a younger preterm-born sibling have lower or higher internalising or externalising behavioural scores compared to children with only term-born siblings.*

QR1-12: Lines 382-384: Differences (or the lack thereof) in what? The authors explained how the results of this study compare to the present literature but failed to provide a takeaway message (does this study add to the current literature or contradict it? What should be the next step?).

**Response:** Thanks for the comment. The points have been addressed as suggested in the revised manuscript (lines 414-420):

*A study on a sample of adult preterm born siblings (N=173) in Canada found no evidence of difference in reported anxiety and mood disorder between the group of adults with preterm siblings and the group with term-born siblings (18). However, the effects were derived from responses by the preterm adults reporting on their siblings’ symptoms. This design may carry important technical (e.g. selection, recall) and emotional biases, in addition to being underpowered. Future studies would benefit from collecting data directly from the siblings themselves.*

QR1-13: Line 389: Avoid using the term "only-child children" as it can lead to confusion. Instead you could use "children without siblings" or "only-children", as done before in the manuscript.

**Response:** Thanks for the comments. This has been done as suggested.

QR1-14: Line 392: Similarly, avoid using the term "not only-child peers". It could be replaced by a simpler version like "children with siblings" or "peers with siblings", as done before.

**Response:** Thank you for the comment. The revised manuscript has been corrected as suggested.

QR1-15: Line 394: The authors are encouraged to describe the mentioned study (39) in more detail as it will add important information to the discussion.

**Response:** Thanks for the suggestion. We have extended this further in the revised manuscript (lines 430-438):

*This study evaluating self-control, interpersonal skills and externalising behaviour between only-children and children with one and more siblings, suggested that children with one or more siblings fared better than their only-child peers in all domains. This study reported that factors such as sex of siblings or birth order had little effect on the association between having siblings versus not and externalising behaviour. It also provided evidence supporting that children with an age gap of three years or less with their siblings had poorer externalising outcomes compared to those with higher age gaps. When it came to self-control, the study reported that closely spaced children exhibited better self-control compared to those with wider age gap.*

QR1-16: Line 395: The results of this study do not suggest that children’s behaviour is affected by a multitude of factors. In detail, this study showed that children with pre-term siblings do not differ in their internalizing or externalizing behavior compared to children with term siblings, though children without siblings show increased internalizing and externalizing behavior compared to children with term siblings. Other factors that might have contributed to the behavioral results were not examined. Please revise this statement to accurately reflect the results.

**Response:** Many thanks for the comment to raise precision and rigor in our manuscript. We have corrected as suggested in the revised manuscript (lines 439-441):

*Our results suggest that having a preterm sibling is not in itself a risk factor for increased internalising and externalising behaviour. Yet, children without siblings had higher externalising and internalising scores than those without a sibling.*

QR1-17: Lines 405-427: The source of behavioral reporting is indeed an important factor that needs to be considered when looking at the results. The authors could show that parents with more than one child have a higher threshold of categorizing their child’s behavior as problematic. However, the authors than argue that parents with only one child will overreport their child’s behavioral problems. While this is an interesting thought, providing a new direction for future research, it should not be mentioned in the abstract (lines 76-77) as this has not been investigated yet.

**Response:** Many thanks for the suggestion. The sentence has been removed from the abstract as suggested.

QR1-18: Line 438-440: Heterogeneity was not investigated in this study, though it should be to examine possible factors that could have led to high heterogeneity. In typical meta-analyses, this is done via meta-regression.

**Response:** We fully agree with the reviewer about this comment. However, there are some barriers that blocked us from doing meta-regression. We only have 4 studies and because we divided our analysis into three age-groups we have data from only two studies in two of the age-groups and data from three studies in the third age-group. There is a rule of thumb that meta-regression should generally not be considered when there are fewer than ten studies in a meta-analysis (e.g. <https://handbook-5-1.cochrane.org/chapter_9/9_6_4_meta_regression.htm>). The small number of studies also blocked us from doing a “leave-one-out” sensitivity analysis. In addition to the small number of studies, we also have a small number of data in the only-child group for which we get high heterogeneity for the model with internalising behaviour at the age-group 5-10 years. The small number of data might produce biased results in the regressions, as we also discussed in the paper, and this might also lead to biased results in meta-analysis and further in meta-regression. The third barrier is the unavailability of specific study-level variables that could be used as potential effect modifiers in a meta-regression. The unavailability is related to multiple reasons including that cohorts might not collected data on such variables or might not harmonised such data for federated analyses. Also, for this project we got a remote access (through DataSHIELD) to a specific set of variables that we included in a project proposal, approved initially by the EU Child Cohort Network and then by the participating cohorts. Access to additional variables is therefore a long-time process.

For all these limitations we therefore decided to report the indicators of heterogeneity (e.g. I squared values) but to not explore its possible factors. We hope that the reviewer understands these barriers. We further expanded the text for the limitations of our study in the discussion section (lines 498-502):

*The heterogeneity observed in the population is potentially created by the age differences within and across the cohorts, in addition to other characteristics. However, we have not been able to further examine the heterogeneity, for example using meta-regression, due to the small number of cohorts and to the unavailability of cohort-level variables.*

QR1-19: Line 445-448: This is an important point that also needs to be mentioned in the methods section (see comments about confounders above).

**Response:** Thanks for the suggestion. We have added this point under Confounders section (lines 244-249):

*Despite evidence of the impact of maternal mental health on internalising and externalising behaviour of children we have not been able to include such variable as a confounder due to a high proportion of missing values. The harmonised data indeed include an indicator for any exposure to relatives such as parents or other individuals close to the child with mental disorders, however the proportion of missing values in this yearly repeated variable ranged from 67.3% in DNBC to 100% in MoBa.*

QR1-20 Lines 463-465: This is indeed a serious limitation, which also needs to be mentioned and discussed in more detail in the discussion section. Would the results change if the group sizes change (e.g., would the significant differences between the only-child and reference groups become non-significant)?

**Response:** We acknowledge an important limitation in the presented results related to the size of the groups. Notwithstanding the fact that the observed effects were consistent in terms of size and directionality across participating cohorts, additional external replication is required to observations can be generalized. To the best of our knowledge, our study plan, as well as the eligibility criteria, should not have introduced a distinct selection. Despite a restricted sample size constrained by our research question there is supportive evidence (the confidence intervals are relatively narrow, allowing us to exclude clinically/practically meaningful differences between siblings of preterm and term children) that the measured effects are robust (low risk of type I or type II errors) but may change in magnitude with large sample sizes.

QR1-21: Lines 475-478: This is a great suggestion: research focus should shift toward regions with high prevalence of preterm birth. Differences in demographics, culture, health care system, etc. could affect behavioral outcomes of children with preterm siblings! The cohorts used for this study were indeed all conducted in high-income countries with excellent health care for parents and children – could this have affected the results?

**Response:** Thanks for this suggestion. We have included the following text in the revised manuscript (lines 536-541):

*This study included only participants living in high-income European countries with good and supportive healthcare and social systems for parents and children, which limits the generalizability of our findings to other populations. Future studies including other regions and countries, where incidence of preterm birth is higher and healthcare systems are weaker, might provide different results to those observed in the current study.*

QR1-22: Lines 488-489: Avoid using the term "mental health problems", use the previously used term “internalising and externalising behaviour” instead to keep the manuscript consistent.

**Response:** This has been corrected in the revised manuscript as suggested by the reviewer.

QR1-23: Lines 489-492: Various suggested factors from the discussion section are missing here (e.g., siblings mental health, sample size of risk group, regions with high prevalence of preterm birth) but should be included.

**Response:** Thanks for the suggestion. We have included the following text in the conclusions section of the revised manuscript (lines 549-553):

*Considering this study’s limitations, we highlight important areas for improvement in future longitudinal studies, such as inclusion of siblings’ mental health, increased sample size of the risk group, inclusion of regions with high prevalence of preterm birth and weaker social and healthcare systems.*

QR1-24: Supplement S3, S4, S6: not mentioned in the text, only in the supplement section (706-712). Please revise the text to include them.
S4: Not found in supplement.
S6 ans S10: The text titles and supplement titles are different. Please revise.

**Response:** Many thanks for this comment. Corrections have been made to supplementary documents and to manuscript body as recommended. Corrections are summarised below:

Supplement 1 corrected to show Cohorts’ info instead of Cohorts info

Supplement 2 is now study design, which has been moved from the main manuscript as suggested by reviewer 2

Supplementary 3 has been mentioned in the text now (lines 311) and line 352 has been corrected, so instead of showing S2 it shows S3

Supplementary 4 mentioned in line 272.

Supplementary 5 mentioned in line 274

Supplementary 6 mentioned in lines 275, 365 and 387

Supplementary 7 has been mentioned in the text and line 568 corrected. So instead of showing S10 it says S7.

- End of responses to reviewer 1 -

Reviewer #2: This study was based on a database of harmonised data from four birth cohorts participating in the European Child Cohort Network. A total of 65,711 term-born children (49% girls) across the four cohorts were grouped as follows: risk group (with younger preterm born siblings; n=427), reference group (with only term siblings; n=12,371); and only-child group (without siblings; n=52,913) to assess differences in internalising and externalising behaviours in siblings as reported by parents on the SDQ or CBCL.

QR2-1: Abstract- well written, however suggest authors reframe conclusion statement re “This finding is consistent with previous reports suggesting overreporting of behavioural problems by parents of an only-child.” as there may be other reasons for over reporting of behavioural problems in one child only parents.

**Response:** Many thanks for this comment, which was also given by Reviewer #1. We have removed this sentence from the abstract.

Introduction- succinct

QR2-2: Methods- several limitations but my main concern is comparing cohorts using two different scales ie SDQ data versus CBCL- can authors please comment on reliability and validity of methods used

**Response:** Indeed, the internalising and externalising scores were measured by different instruments and at multiple data collection waves across the cohorts. To overcome this limitation, we standardised the raw scores using z-score transformations at each age and for each cohort separately. In such cases, a score for a child can be comparable with the scores of its peers at the same age independently of the instrument used. In the revised manuscript (lines 204-225) we now have the following text:

*Internalising and externalising behaviours were measured using one of two well-established parent-report instruments: the Strengths and Difficulties Questionnaire (SDQ; DNBC and NINFEA) (29), or the Child Behaviour Checklist (CBCL; GenR and MoBa) (30). The SDQ contains 25‐items measured on a 3‐point Likert scale (“not true”, “somewhat true”, “certainly true”) containing five subscales: Emotional Problems, Conduct Problems, Hyperactivity, Peer Problems, and Pro‐social behaviour. Internalising scores are calculated by combining the Emotional Problems and Peer Problems subscales, while externalising scores are calculated by combining the Conduct Problems and Hyperactivity subscales. The CBCL contains 99‐items rated on a 3‐point Likert scale (“not true”, “sometimes true”, “often true”), containing eight subscales: Delinquent Behavior, Aggressive Behavior, Withdrawn, Somatic Complaints, Anxious/Depressed, Social Problems, Thought Problems, and Attention Problems. Internalising scores are calculated by combining the Withdrawn, Somatic Complaints, and Anxious/Depressed subscales, while externalising scores are calculated by combining the Delinquent and Aggressive Behaviours.*

*To allow statistical comparability across instruments we z-standardized the internalising and externalising scores by age according to the term born children’s scores within each cohort before the main analysis. Z-scores have been calculated by subtracting from each individual raw score (internalising or externalising) the mean value and dividing by the standard deviation of the raw scores of all term born children in the cohort. This transformation standardises the scores to a distribution with mean equal to 0 and standard deviation equal to 1. This helps overcome scaling differences across the harmonised dimensional scores within the respective age-bands of the CBCL and SDQ.*

QR2-3: Results - cohorts are stratified into age groups- Do we know age at which the focal child had sibling and temporal association between the event and the assessment timings

**Response:** Many thanks for this comment and question. Indeed, it would have been of added value to investigate the at which age the focal child had a sibling. Unfortunately, we do not have the relevant variables available in our dataset, and therefore have not been able to conduct this additional analysis. We have addressed this in the discussion (lines 455- 460):

*In addition, as suggested in previous studies, the age gap between siblings might influence the associations, however, in the current study* we have not been able to examine the effect of the age difference between the focal children and the preterm children (39*). Equally, we have not been able to investigate the association between the age of the focal child at time of birth of the preterm sibling and the outcomes.*

discussion- authors do discuss the limitations.

Conclusion- fair

QR2-4: Whilst there are multiple limitations, results do contribute to the limited evidence on the subject.

**Response:** Many thanks for this. We agree and have acknowledged the study limitations accordingly.

- End of responses to reviewer 2 -

Reviewer #3:

QR3-1: The authors state that “it has been found that family stress levels remain elevated seven years after the birth of a preterm child, (11). Thus, it can be hypothesized that siblings may also be affected by having a preterm born sibling”. Can authors add a sentence or two to describe how elevated family stress would impact the children?

**Response:** Many thanks for the suggestion. We have added a short description to explain how elevated parental stress might impact the children. In the revised manuscript (lines 95-97) we have now the following text:

Families with children born preterm (before 37 weeks of gestation) may experience changes in their home environment stemming from the health care needs of the preterm child. Short- and long-term impacts of preterm birth on parents, such as stress and depression, have been extensively studied (10–12). For instance, it has been found that family stress levels remain elevated seven years after the birth of a preterm child (11). *Such changes in mental health and psychosocial stress may impact parenting, which, in turn, may affect the children’s behaviour*. Thus, it can be hypothesised that siblings may indirectly be affected by having a preterm *born sibling.*

QR3-2: The study has not examined several important confounding factors that could have influenced the externalizing and internalizing symptoms. These include
o socio-economic status – families from lower SES will have fewer resources to mange the needs of a pre-term child, which may lead to increased family stress and behavioural issues in the older sibling
o parents’ marital status – single mothers will have greater difficulty in providing care to the older sibling while caring for a pre-term child
o age of the focal child at the birth of the preterm sibling – children who were older at the time of the birth of the pre-term sibling may have better coping skills to adjust to the demands of having a pre-term sibling
o whether the preterm sibling required long term hospitalized care or had medical needs that required frequent hospitalizations– if the mother had to accompany the pre-term sibling in hospital for long periods, this would have greatly impacted the mental health of the sibling
o family support – family support (e.g., from extended family) may influence how the mother coped with the needs of the preterm child while caring for the older sibling.

A separate paragraph/paragraphs needs to be added to the discussing these confounding factors, reflecting on previous literature and how they may have influenced the results of the study. The limitations section should clearly indicate that the validity of the results is impacted by these confounding factors. (Line 512)

**Response:** Many thanks. We have addressed this comment by further discussing the confounding factors in the Discussion section. The following text is now included in the revised manuscript (lines 441-464):

*In this study we adjusted for parental education (an indicator of socio-economic status), smoking and alcohol use during pregnancy. These variables have been shown to be associated with preterm birth and with internalising and externalising behaviour, However, results of unadjusted and adjusted analysis suggest that these confounders did not affect the results observed. However, the increased internalising and externalising behaviour in children without siblings compared to children with term siblings, suggests that other factors not examined in the current study might have contributed to the behavioural results observed. For example, studies investigating mental health outcomes of children whose siblings are affected by autism or chronic diseases have suggested that birth order and the higher externalising in the sibling may play a significant role in the outcome of the focal child (40,41). In our study, the focal children in the risk group are older than their preterm sibling, and we were able to investigate whether the arrival of the preterm child affected behaviour of the focal child. However, we have not been able to test whether internalising and externalising scores of the term-born children were comparable to scores of their preterm siblings. In addition, as suggested in previous studies, the age gap between siblings might influence the associations, however, in the current study we have not been able to examine the effect of the age difference between the focal children and the preterm children (39). Equally, we have not been able to investigate the association between the age of the focal child at time of birth of the preterm sibling and the outcomes. Other variables that could explain the internalising and externalising behaviour scores, such as mother’s marital status, information on short- or long-term hospitalization and medical complications of the preterm child, the available support network, were not available. Considering all the above, the validity of the results of our study is impacted by the variables available.*

- End of responses to reviewer 3 -
