# Supplementary material for: Internalising and externalising behaviour in siblings of children born preterm Preterm birth: Internalising and externalising behaviour of siblings
Source: PLOS Ment Health. 2025 Jun 11;2(6):e0000334. doi: 10.1371/journal.pmen.0000334 (PMC12798436; doi:10.1371/journal.pmen.0000334)
Supplement: Attachment — Submitted filename: PMEN-D-24-00079_R2 Response letter.docx [file pmen.0000334.s010.docx]

Dear Editor,

We would like to thank the reviewer 4 for their comments and suggestions. We have revised the manuscript carefully in response to the comments and suggestions. Below we respond to the reviewer’s comments and describe the changes introduced in the revised manuscript.

We hope that the responses provided in this document and in the manuscript positively address the reviewer’s comments and suggestions. However, please do not hesitate to contact us should you have any questions or further suggestions.

Kind regards,

Wnurinham Silva (on behalf of the authors team)

Note: Line numbers refer to the clean version of the manuscript.

Reviewer # 4

Abstract
**Q1.** The abstract can be made more concise by focusing on the key findings and their implications, while omitting less critical details (e.g., specific cohort statistics or methods not central to the conclusion).

**Response:** Many thanks for your comment. We reviewed the abstract and have attempted to make it more concise.

**Q2**. Clarify the hypothesis and ensure it is aligned with the results and conclusion.

**Response:** Many thanks. We have changed the hypothesis in the abstract to make it clearer and to align with the results and conclusion.

Introduction
**Q3**- Provide a more in-depth explanation of why sibling dynamics in the context of preterm births are significant for mental health research.

**Response:** Thank you for this comment. We have added a brief explanation on why term born children with preterm born siblings are important for mental health research. Please see below and lines 118- 121.

*Considering that children with preterm born siblings are potentially at increased risk of experiencing mental health disorders, but grossly overlooked, further studies are needed to clarify this relationship. Such studies could provide invaluable evidence of need of targeted interventions to this group.*

**Q4**- Expand the discussion of existing studies, particularly those with conflicting findings, to better justify the study's hypothesis.

**Response:** Thank you for the suggestion. We have briefly expanded the discussion of existing studies. Please see lines 109-113 and below.

*Two studies investigating the impact of having a preterm born sibling reported no difference in behavioural disorders between term born children with preterm born siblings and those with term -born siblings (17,18). However, another study found that the group of term-born children with preterm born siblings had more behavioural disorders compared to those with term-born siblings (19).*

**Q5**- Clearly articulate the research hypothesis and its rationale, ensuring alignment with the study design and outcomes.

**Response:** Many thanks for the suggestion. Please see lines 124-126.

Methods
**Q6**- While the harmonization across cohorts is a strength, provide additional details on how differences in data collection (e.g., timing, instruments) were addressed.

**Response:** All the confounders have been measured at baseline (e.g. at birth) and therefore there are no differences in the timing of measurements. Two of them (maternal smoking and alcohol intake) are binary variables (yes/no), one is continuous (maternal age) and another one (maternal education) is categorical with three levels. Details about these variables and how maternal education is harmonised can be found in lines 244-256. The outcome variables are the only ones that had differences at the timing and instruments across the cohorts. To address the different instruments used we standardised the raw scores using z-score transformations. This helps overcome scaling differences across the harmonised dimensional scores. Details about the outcomes and their standardisation can be found in lines 213-235. To address the differences in timing (i.e. collection of the outcomes at different ages across cohorts) we analysed the outcome variables in three age groups created based on data availability and developmental stages: ages equal and less than 4 years (early childhood), ages from 5 to 10 years (mid-childhood), and ages from 11 to 14 years (pre-adolescence and early adolescence). In each age group, if a child had multiple scores, we used the latest available. Details about the age groups can be found in lines 235-239.

**Q7**-Justify the exclusion of maternal mental health data due to missingness and discuss its potential impact on findings.

**Response:** We were not able to include maternal mental health as a confounder due to a high proportion of missing values. The harmonised data include an indicator for any exposure to relatives such as parents or other individuals close to the child with mental disorders, however the proportion of missing values in this yearly repeated variable ranged from 67.3% in DNBC to 100% in MoBa. We have added extended the discussion in lines 516-525, as below:

*Maternal (and the other parent) mental health could confound and/or modify the association presented in this research; but, the magnitude and the directionality of the change upon the reported effects would be hard to estimate from the literature. The parental health status may indeed act upon the exposure (i.e. having a sibling born preterm) and the outcome (i.e. alteration on internalizing or externalizing signals) via environmental and genetic predispositions. The association on both the exposure and the outcome can be relatively balanced (shared environment confounders) or un-balanced (modifying either the propensity of the exposure to change the outcome and/or the proportion of cases). We recommend continuing multi-cohort studies, and, combining it with other clinical designs to continue triangulating the effects of parental and sibling upon a child’s mental health.*

**Q8**-Elaborate on how heterogeneity between cohorts was managed, especially in the meta-analysis.

**Response:** Many thanks for the comment. Due to some barriers, we were not able to further explore the observed heterogeneity and therefore we mentioned that as a limitation of our study. For example, we were not able to run meta-regression. We only have 4 studies and because we divided our analysis into three age-groups we have data from only two studies in two of the age-groups and data from three studies in the third age-group. There is a rule of thumb that meta-regression should generally not be considered when there are fewer than ten studies in a meta-analysis (e.g. https://handbook-5-1.cochrane.org/chapter_9/9_6_4_meta_regression.htm). The small number of studies also blocked us from doing a “leave-one-out” sensitivity analysis. In addition to the small number of studies, we also have a small number of data in the only-child group for which we get high heterogeneity for the model with internalising behaviour at the age-group 5-10 years. The small number of data might produce biased results in the regressions, as we also discussed in the paper, and this might also lead to biased results in meta-analysis and further in meta-regression. The third barrier is the unavailability of specific study-level variables that could be used as potential effect modifiers in a meta-regression. The unavailability is related to multiple reasons including that cohorts might not collected data on such variables or might not harmonised such data for federated analyses. Also, for this project we got remote access (through DataSHIELD) to a specific set of variables that we included in a project proposal, approved initially by the EU Child Cohort Network and then by the participating cohorts. Access to additional variables is therefore a long-time process. For all these limitations we therefore decided to report the indicators of heterogeneity (e.g. I squared values) but to not explore its possible factors. We hope that the reviewer understands these barriers. We include the text for this limitation of our study in the discussion section (lines 504-508).

**Q9**- Highlight steps taken to mitigate the effects of small sample sizes in the risk group.

**Response:** Many thanks for the recommendation. The method of individual participant meta-analysis was used to mitigate the effects of small sample sizes in the risk group. Both the two-stage analysis and the one-stage analysis, which was used in a sensitivity test, are combining the effects from the data from all cohorts together and therefore our analysis is based on a larger sample than the case of analysing the data from each cohort separately. We have highlighted this in the discussion, please see lines 542-547.

*However, the method of individual participant meta-analysis was used to mitigate the effects of small sample sizes in the risk group. In the two-stage meta-analysis we fitted a fixed-effects linear regression model in each cohort separately and then combined the cohort-specific estimates using random-effects meta-analysis. In the one-stage meta-analysis considers all cohorts as one by pooling the data from all cohorts together and thus maximizing their sample size.*

Discussion
**Q10-** Strengthen comparisons with existing studies, particularly in explaining discrepancies.

**Response:** Many thanks, we have attempted to strengthen comparison with existing studies by highlighting discrepancies.

**Q11-** Discuss potential biases (e.g., parental reporting) and their impact on the findings more comprehensively.

**Response:** Many thanks. This has been addressed in lines 552-556.

*In addition, as indicated above, both CBCL and SDQ questionnaires are parent-reported, which may be at risk of reporting bias. However, a study has suggested that self and parent reported SDQs were equally effective at predicting presence of psychiatric disorders (51). Yet, future studies would benefit from including multiple sources report.*

**Q12-** Expand on the implications of the findings for clinical practice and policy, especially regarding interventions for siblings of preterm children.
**Response:** Many thanks, we have done this in lines 576-577, whilst trying to stay succinct.

**Q13-** Provide concrete suggestions for addressing identified gaps, such as collecting direct sibling-reported data or including larger sample sizes.

**Response:** Many thanks for this suggestion. Concrete suggestions have been provided for the identified gaps such as in lines 523-525 and 555-556.

Conclusion
**Q14-** Summarize the findings and their implications succinctly, avoiding repetition from other sections.

**Response:** Many thanks for this suggestion. We have attempted to summarise as much as possible, without repetition.

**Q15-** Briefly reiterate the key limitations to provide context to the conclusion.

**Response:** Thanks for this suggestion. We have addressed this in lines 570-573.

**Q16-** Recommendations: End with clear recommendations or calls to action for future studies or policy changes.

**Response:** Many thanks for this recommendation. We have closed the conclusion with recommendations. Please see lines 577-580.

In addition, we have added information to the acknowledgments section, lines 594-600.
