# Supplementary material for: Internalising and externalising behaviour in siblings of children born preterm Preterm birth: Internalising and externalising behaviour of siblings
Source: PLOS Ment Health. 2025 Jun 11;2(6):e0000334. doi: 10.1371/journal.pmen.0000334 (PMC12798436; doi:10.1371/journal.pmen.0000334)
Supplement: Attachment — Submitted filename: Response letter 020425.docx [file pmen.0000334.s011.docx]

| **To the editor of PLOS Mental Health** | Wnurinham Silva  Doctoral Researcher  Research Unit of Population Health  Faculty of Medicine  University of Oulu  Oulu, Finland  02.04.2025 |
| --- | --- |

**RE: Response letter**

**Manuscript number:** **PMEN-D-24-00079R2**

**Title: Internalising and externalising behaviour in siblings of children born preterm**

Dear Editor,

We hope that this letter will find you well.

We would like to thank the reviewer 5 for their constructive comments and suggestions. We have revised the manuscript carefully in response to the comments and suggestions. Below we respond to the reviewer’s comments and describe the changes introduced in the revised manuscript.

We trust that the responses we are hereby providing in this document and in the manuscript will address the reviewer’s comments and suggestions.

However, please do not hesitate to contact us should you have any questions or further suggestions.

Kind regards,

Wnurinham Silva (on behalf of the authors)

Note: Line numbers refer to the clean version of the manuscript.

You will find below the point-by-point responses to reviewer 5.

Reviewer # 5

**Q1.** The small number of children in the risk group (with preterm siblings) reduces the statistical power, which may limit the reliability of the results for this group. Acknowledging this limitation more explicitly and discuss how it may affect the generalizability of the findings. Future studies could aim to include a larger sample of children with preterm siblings.

**R:** Many thanks for this suggestion. We have tried to acknowledge this as a limitation and have briefly discussed how findings might not be generalizable. Please see lines 560-561 and 567-574 (below).

*Yet, we acknowledge that our sample size in the risk group and subsequently the potential loss of reliability of our findings in this group are important limitations. Similarly, the countries included in this study have relatively low percentage of preterm birth, therefore our findings might not be generalizable to other countries with much higher prevalence of preterm birth. The study is unfortunately limited by the small sample size in the risk group and we therefore recommend that future studies with a larger sample size will be needed to replicate and help a better understanding of the observed associations.*

**Q2.** The different ages at which internalising and externalising scores were collected across cohorts may introduce heterogeneity and biases in results. Considering providing more detailed explanations of how cohort-specific differences in measurement age were handled and discuss how this could influence the findings. Cohort-specific analyses may also add clarity.

**R:** Many thanks for this very important suggestion. We have added more information about how we handled the data collection at different ages in different cohorts (lines 239-241) and discussed possible implications (lines 507-516).

**Q3.** The lack of maternal mental health data (a known confounder) due to high missingness could bias the results. It would be helpful to address how the missing maternal mental health data might impact the results and whether future research could include better tracking of this variable.

**R:** Many thanks for this comment and suggestion. We currently discussed the potential impact of the missing maternal mental health data on our results in lines 521-535.However, as suggested we have added a brief suggestion for future research to keep better tracking of maternal mental health variables (Please see lines 531-535).

**Q4.** This manuscript focuses on sibling status but does not delve deeply into other factors, such as family dynamics, parental health, or support systems, which could also contribute to behavioural outcomes. Those robustness analyses should be added.

**R:** Many thanks for this comment. We agree that the above-mentioned measures may help testing the robustness of the finding and could be a relevant addition to help inferring the problem at hand.

We would like to acknowledge important points that makes it challenging for us to implement this request. All the work presented is done with participating cohorts of the EU Child cohort network (<https://euchildcohortnetwork.eu>) with federated, non-disclosive methods (DataShield). One key added value, which also brings some limitations, is the fact that it relies on a large effort by all cohort to harmonize and catalogue their data to allow pooled and individual patient level meta-analyses. We fully agree that harmonized or comparable measure of family dynamics, parental health, or support systems will be of great added value. Performing such analysis would require us to harmonize such factors across cohorts which will lead to a very substantial effort and a lengthy process.

In the current version, we have acknowledged that other factors might explain the results observed (454-456) and provided just a few examples of other possible factors in lines 456-496, including parental health and family dynamics in lines 490-491. We have added the following sentence in the strength and limitations (lines 535-543):

*Additionally, it must be acknowledged that we could not perform robustness analyses to infer the contribution of family dynamics, parental health or the role of the support systems on later outcomes. These measures were unfortunately missing from the data of the EU Child cohort network (https://euchildcohortnetwork.eu) and we recommend future harmonization across cohorts to include factors such as adverse childhood experiences (ACEs), parental relationships, schooling and education systems in the databases. We also recommend developing and linking longitudinal measures of maternal, paternal and child health data to further model their dynamics across the life-course.*

**Q5.** The manuscript uses complete case analysis but does not provide sufficient detail on how missing data was handled across the cohorts.

**R:** Many thanks for this comment. We would like to clarify that all cohorts provided harmonised data and that in the current study we used complete case (cohort level and not study level). We have tried to clarify this in the manuscript in lines 275-276.

**Q6**. Study heterogeneity should be examined for the between-cohort condition. The manuscript reports pooled results without sufficiently discussing how the findings may vary across different cohorts.

**R:** Many thanks for this comment. We have extended the discussion on heterogeneity to address possible variations across the included cohorts. Please see lines 512-516.

**Q7.** A significant confounder - the potential bias from parental reporting of behaviours, should be considered. Parental reports may be biased, especially in families with only-children, where parents might report more behavioural issues.

**R:** Many thanks for this very important comment, which has been raised by previous reviewers and which we have tried to address as well as possible in previous versions. Please see lines 472-488 and below.

*It has also been shown that the source of the behavioural reports may influence the findings. Most of the above cited studies (17,19,38,39), including the current study, share the study design whereby the outcomes are based on parental reports. Previous research suggests that parents’ and children’s reports on behaviour tend to vary, with parents more likely to report lower rates of child symptoms than children themselves (40–42). Factors such as the children’s sex and age, type of outcome, socio-economic characteristics and parent-child relationship have been associated with discrepancies between parent and child reports (43). It has also been suggested that the presence of a sibling in the household is associated with mothers reporting less behavioural problems in their children (42). The authors of that paper hypothesized that the presence of other children in the family may raise the threshold for which mothers consider her child’s behaviour as problematic (42). Therefore, it is possible that the higher scores observed in the only-child group could be a result of such response from mothers, biasing maternal reports. Hence, one may also speculate that parents of preterm children, who often have behavioural and neurodevelopmental difficulties, may be biased towards under-reporting the internalising and externalising problems of their term-born children.*

**Q8.** I am not sure whether this data contain information for the potential influence of the age gap between siblings on the behavioural outcomes. If gathering this data, this age gap should be modelled into the statistical analysis, given it has well-acknowledged for roles in the within-family mental health.

**R:** Many thanks for this comment, which has been also raised by previous reviewers. We recognise this would be an important analysis, however, we are limited by data availability. This has been addressed as a limitation in lines 463-468, and as below:

*In addition, as suggested in previous studies, the age gap between siblings might influence the associations, however, in the current study we have not been able to examine the effect of the age difference between the focal children and the preterm children (37). Equally, we have not been able to investigate the association between the age of the focal child at time of birth of the preterm sibling and the outcomes.*
